# Sub-Nyquist SAR Based on Pseudo-Random Time-Space Modulation

**DOI:** 10.3390/s18124343

**Published:** 2018-12-09

**Authors:** Wenjiao Chen, Chunsheng Li, Ze Yu, Peng Xiao

**Affiliations:** 1 School of Electronics and Information Engineering, Beihang University, Beijing 100083, China; chenwenjiao008@163.com (W.C.); 00178@buaa.edu.cn (C.L.); 2 Department of Applied Science and Frontier Technology, Qian Xuesen Laboratory of Space Technology, Beijing 100094, China; xiaopeng@qxslab.cn

**Keywords:** spaceborne synthetic aperture radar (SAR), sub-Nyquist, noisy-channel coding theorem, channel capacity, sparsity, pseudo-random space-time modulation

## Abstract

Sub-Nyquist sampling technology can ease the conflict between high resolution and wide swath in a synthetic aperture radar (SAR) system. However, the existing sub-Nyquist SAR imposes a constraint on the type of the observed scene and can only reconstruct the scene with small sparsity (i.e., number of significant coefficients). The information channel model of microwave imaging radar based on information theory, in which scene, echo, and the mapping relation between the two correspond to information source, sink, and channel, is built, and noisy-channel coding theorem explains the reason for the aforementioned under this model. To allow the wider application of sub-Nyquist SAR, this paper proposes sub-Nyquist SAR based on pseudo-random space-time modulation. This modulation is the spatial and temporal phase modulation to the traditional SAR raw data and can increase the mutual information of information source and sink so that the scenes with large sparsity can be reconstructed. Simulations of scenes with different sparsity, e.g., an ocean with several ships and urban scenes, were run to verify the validity of our proposed method, and the results show that the scenes with large sparsity can be successfully reconstructed.

## 1. Introduction

Sub-Nyquist sampling technology [1,2,3,4,5,6,7], in which the sampling frequency is smaller than the Nyquist sampling rate [8], was proposed because there is redundancy in any information [9]. This technology can be applied in various fields, e.g., wireless communication [10] and synthetic aperture radar (SAR) [11,12,13,14,15,16,17]. Synthetic aperture radar takes samples of signals reflected from an illuminated scenario/scene to obtain a high-resolution image after processing at all times and in all weather conditions [18,19]. In a traditional SAR system, the pulse repetition frequency (PRF) should satisfy the Nyquist sampling theorem and exceed the Doppler bandwidth to avoid spectrum aliasing, and the Doppler bandwidth is inversely proportional to the azimuth resolution. This means that the better the resolution, the higher the PRF. Additionally, the echo must be completely received within one pulse repetition interval, i.e., the reciprocal of the PRF. This means that the larger the swath, the lower the PRF. Therefore, high resolution requiring high PRF and wide swath requiring low PRF are a contradiction [18,19]. When sub-Nyquist sampling is adopted on the azimuth dimension, the contradiction between high resolution and wide swath can be eased under compressive sensing (CS) theory.

The restricted isometry property (RIP) is a sufficient and necessary condition for successful reconstruction in CS theory [20]. The RIP implies that the sparser the scene is, the more easily the RIP is satisfied. However, approximately certifying the RIP is challenging [21]. In the SAR system, the RIP is usually satisfied by random sub-Nyquist sampling on the azimuth dimension, and this sampling method is achieved by randomly transmitting a smaller number of pulses or randomly selecting from the Nyquist samples [7,15]. Under this sub-Nyquist sampling method, the reconstructed scenes are required to be sparse according to CS theory [20,22], and most references [7,11,15,23] directly assume that the reconstructed scene is sparse when a small number of very strong reflectivity areas exist in a darker background. Because the azimuth reconstructed matrix is decided by the Doppler movement, SAR has the limitation of application on the type of the observed scene only by random sub-Nyquist sampling method. In this paper, we explain the reason for this constraint from the perspective of information theory and relieve this constraint to make the scene more widely applicable. Additionally, greedy algorithms [24,25], *l*_1_ relaxation methods [26,27,28,29], and Bayesian-based methods [30,31,32] can be used under the assumption of satisfying the RIP. This paper mainly adopts *l*_1_ relaxation methods with small recovery error compared with the other two algorithms [33].

Receiving the raw data from the observed scene is essentially the procedure of information transferring in the microwave imaging radar system [34,35]. Therefore, the information channel model of the microwave imaging radar is built to analyze the transferring information entropy from the perspective of information theory, and the observed scene, the raw data and the mapping relation between the two correspond to information source, sink, and channel. The carrier of transferring information is information channel. Channel capacity is a quantitative index which evaluates the quality of information channel. Under this model, noisy-channel coding theorem indicates that all information is nearly error-free transferred in one coded way, while the transferring information entropy is smaller than the channel capacity, otherwise reliable transferring cannot be achieved [36]. This theorem explains the necessary requirement of the reconstruction and the reason for the constraint on the sparsity of scene.

To relieve the constraint on the type of the observed scene in the existing sub-Nyquist SAR system so that the scene with large sparsity can be reconstructed, this paper proposes sub-Nyquist SAR based on pseudo-random space-time modulation. Under the information channel model of the microwave imaging radar based on information theory, this modulation is the spatial and temporal phase modulation to the traditional SAR raw data and can increase the mutual information of information source and sink so that more information about scene can be achieved. After the pseudo-random space-time modulation, the sub-Nyquist SAR system can also reconstruct the scene with large sparsity. This system lays the foundation to achieve a high-resolution and wide-swath SAR system.

The rest of this paper is organized as follows. Section 2 presents the constraint on the type of the observed scene in the existing sub-Nyquist SAR system. In Section 3, the information channel model to the microwave imaging radar based on information theory is established, and noisy-channel coding theorem is used to explain the reason for the constraint under this model. In Section 4, the pseudo-random space-time modulation is applied to the traditional SAR raw data to relieve this constraint, and the compressive reflector antenna (CRA) is taken as the carrier of this modulation. Section 5 proposes the sub-Nyquist SAR based on pseudo-random space-time modulation, which is described from four aspects: the choice of the sampling method, the echo signal model, the reconstruction method, and the reconstruction performance. The simulation results are presented in Section 6, and Section 7 concludes the paper.

## 2. SAR Image Reconstruction Based on CS

After removing carrier frequency through quadrature demodulation, the SAR raw data for point targets can be expressed as

(1) $$s_{c}\left(\tau ,\eta \right)=\sum_{x_{i},y_{i}}^{}\sigma_{i}W_{i}\left(\tau ,\eta \right)\exp\left\{j\pi K_{r}{\left[\tau -\frac{2R_{i}(\eta )}{c}\right]}^{2}-\frac{j4\pi R_{i}(\eta )}{\lambda }\right\}+n\left(\tau ,\eta \right),$$

where τ and η are the fast time along the range and the slow time along the azimuth, respectively, $\sigma_{i}$ and $W_{i}\left(\tau ,\eta \right)$ are the backscattering cross-section and the weighting pattern corresponding to the target at $\left(x_{i},y_{i}\right)$, $x_{i}$ and $y_{i}$ denote the azimuth and range coordinates, respectively, $K_{r}$ denotes the frequency modulation rate of the pulse. $R_{i}(\eta )$ represents the distance between the radar and the point target at the slow time η. c is the speed of light, λ is the wavelength, and n(τ,η) denotes the system noise.

For moderating the contradiction between high-resolution and wide-swath, in this paper sub-Nyquist sampling is implemented along the azimuthal dimension in this paper. Accordingly, SAR image reconstruction includes three steps: range compression based on matched filtering, range cell migration correction (RCMC), and azimuth reconstruction from sub-Nyquist samples based on CS. After implementing range compression and RCMC to Equation (1), the signal at a certain range cell is represented by:

(2) $$s_{c}\left(\tau_{0},\eta \right)=\sum_{x_{i},y_{i}}^{}\sigma_{i}W_{i}\left(\tau_{0},\eta \right)Tr\times \sin c\{K_{r}T_{r}(\tau_{0}-\frac{2R_{i}(\eta -\eta_{ci})}{c})\}\times \exp\left\{-\frac{j4\pi R_{i}(\eta -\eta_{ci})}{\lambda }\right\}+n\left(\tau_{0},\eta \right),$$

where $\eta_{ci}$ is the beam center crossing time for the target $\left(x_{i},y_{i}\right)$. Equation (2) can also be expressed as the vector-matrix form

(3) $$s_{N\times 1}=D_{N\times M}\sigma_{M\times 1}+n_{N\times 1},$$

where N is the number of samples on the azimuthal dimension, and M is the number of resolution cells at the certain range cell in the observed scene, $s_{N\times 1}={\left[s_{c}\left(\tau_{0},\eta_{1}\right),s_{c}\left(\tau_{0},\eta_{2}\right),\cdots ,s_{c}\left(\tau_{0},\eta_{N}\right)\right]}^{T}$, $\sigma_{M\times 1}={\left[\sigma_{1},\sigma_{2},\cdots ,\sigma_{M}\right]}^{T}$, $D_{N\times M}={\left\{D_{i}(\tau_{0},\eta_{n})\right\}}_{n=1,i=1}^{N,M}$, $D_{i}\left(\tau_{0},\eta_{n}\right)=W_{i}\left(\tau_{0},\eta_{n}\right)\cdot Tr\cdot \exp\left\{-j4\pi R_{i}(\eta_{n}-\eta_{c_{i}})/\lambda \right\}$, and $n_{N\times 1}={\left[n\left(\tau_{0},\eta_{1}\right),n\left(\tau_{0},\eta_{2}\right),\cdots ,n\left(\tau_{0},\eta_{N}\right)\right]}^{T}$.

To exactly reconstruct $\sigma_{M\times 1}$ from the undetermined system (Equation (3)) for which the unknown number M is larger than the known number N, the RIP of the matrix $D_{N\times M}$ should be satisfied. Assuming that Λ⊆{1,2,⋯,M} is an index subset with S elements and 1≤S≤M, the RIP is quantitatively denoted as [20] for all $\sigma_{\Lambda }\in {\mathbb{C}}^{S}$:

(4) $$\left(1-\delta_{S}\right){\left\|\sigma_{\Lambda }\right\|}_{2}^{2}\leq {\left\|D_{\Lambda }\sigma_{\Lambda }\right\|}_{2}^{2}\leq \left(1+\delta_{S}\right){\left\|\sigma_{\Lambda }\right\|}_{2}^{2},$$

where the restricted isometry constant $\delta_{S}\in \left(0,1\right)$ is a small quantity, ${\left\|\cdot \right\|}_{2}$ is *l*_2_-norm of vector [37], $D_{\Lambda }$ denotes the sub-matrix formed from the columns of D indexed by Λ, and $\sigma_{\Lambda }$ denotes the sub-vector formed from the rows of σ indexed by Λ. The RIP essentially explains that the distance between two signals Dσ and $D\sigma^{\prime }$ is proportional to the distance between the two signals σ and $\sigma^{\prime }$, it can guarantee the exact reconstruction. The sparsity S, the non-zero number of the unrecovered signal $\sigma_{M\times 1}$, is introduced to express the RIP definition. The fewer the number of non-zero elements, the sparser the signal is and the more easily the RIP is satisfied. Additionally, the improvement of RIP condition contributes to enlarging the sparsity so that the reconstructed scene is not only limited to the sparse type.

The RIP implies that randomness plays a crucial role in the reconstructed matrix $D_{N\times M}$. Usually $D_{N\times M}$ has the two following compositional forms [22]: (1) all variables of the matrix obey one certain random distribution; and (2) the rows of the matrix are randomly selected from an orthogonal basis. The azimuthal signal in the SAR system is decided by the Doppler movement between the radar and the scene so that the elements of $D_{N\times M}$ are the Doppler signal, and $D_{N\times M}$ under the Nyquist sampling method is an orthogonal basis. Therefore, if sub-Nyquist samples are randomly selected from the Nyquist samples, the scene may be recovered according to the Form 2. Under the assumption of satisfying the RIP, three categories of the reconstructed algorithm can be used. The first is greedy algorithms, e.g., orthogonal matching pursuit (OMP) [24] and thresholding [25]; the second is *l*_1_ relaxation methods, e.g., the Dantzig selector [26] and basis pursuit denoising (BPDN) [27,28,29]; and the third is Bayesian-based methods, which include maximum a posteriori (MAP) estimation and Hierarchical Bayesian framework [30,31,32]. The greedy method is faster than the other two methods in term of the recovery time, *l*_1_ relaxation method performs better in term of small recovery error, Bayesian method balances between small recovery error and short recovery time [33]. This paper mainly adopts *l*_1_ relaxation methods with small recovery error. The optimization equation of Equation (3) without matrix subscript is

(5) $$\hat{\sigma }=\underset{\sigma }{\arg\min}\left\{{\left\|s-D\sigma \right\|}_{2}^{2}+\gamma {\left\|\sigma \right\|}_{1}\right\},$$

where γ is the regularization factor. The first item ${\left\|s-D\sigma \right\|}_{2}^{2}$ guarantees the recovery accuracy, the second item ${\left\|\sigma \right\|}_{1}$ ensures the sparsity of the recovered scene, and γ balances between the recovery accuracy and the sparsity. The parameter γ, an experience value, is selected by minimizing the reconstructed error for each application.

**Figure 1:**
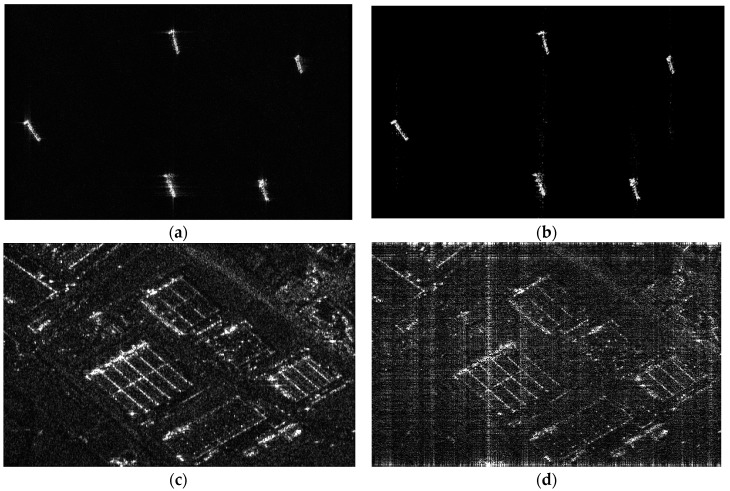
(**a**) Reconstructed result of ocean containing ships from Nyquist samples based on chirp scaling; (**b**) Reconstructed result of ocean containing ships from sub-Nyquist samples based on compressive sensing (CS); (**c**) Reconstructed result of an urban scene from Nyquist samples based on chirp scaling; (**d**) Reconstructed result of an urban scene from sub-Nyquist samples based on CS.

Two TerraSAR-X images, i.e., ocean containing ships with 0.8% sparsity level, and an urban scene with 25% sparsity level, were selected. The sparsity level is percent of significant coefficients in the signal. Their reflectivity functions were used to simulate raw data in Figure 1, and the simulated parameters are shown in Table 1. The results in Figure 1a,c are reconstructed with Nyquist samples based on a chirp scaling algorithm [38]. The results shown in Figure 1b,d are reconstructed with sub-Nyquist samples obtained according to the abovementioned Form 2, and the reconstructed algorithm is based on the optimization equation of Equation (5), the corresponding parameters γ are about 100 and 1, respectively. From the simulation results, we can see that most details of ships on the ocean in Figure 1b are maintained, but the urban scene in Figure 1d is not successfully reconstructed. The explained reason that the reconstructed results with the same simulated parameters are different may be that the sparsity of urban scene does not satisfy the RIP condition so that it is not successfully reconstructed. It is challenging to quantitatively certify the RIP [21]. The simulated results also show that this sub-Nyquist SAR system has constraint on the scene type. The reason for the unsuccessful reconstruction is explained in Section 3.

## 3. Information Channel Model of Microwave Imaging Radar Based on Information Theory

Receiving the echo from the observed scene in the microwave imaging radar system is actually the procedure of information transferring [34,35]. Information transferring is a major field of information theory and transferred information can be quantitatively calculated by the concept of entropy. Scenes with different sparsity have different information entropy. The sparser the scene, the smaller the information entropy. To quantitatively analyze this procedure of information transferring, an information channel model of microwave imaging radar model based on information theory was built and the corresponding relationship between the two should be established.

**Figure 2:**
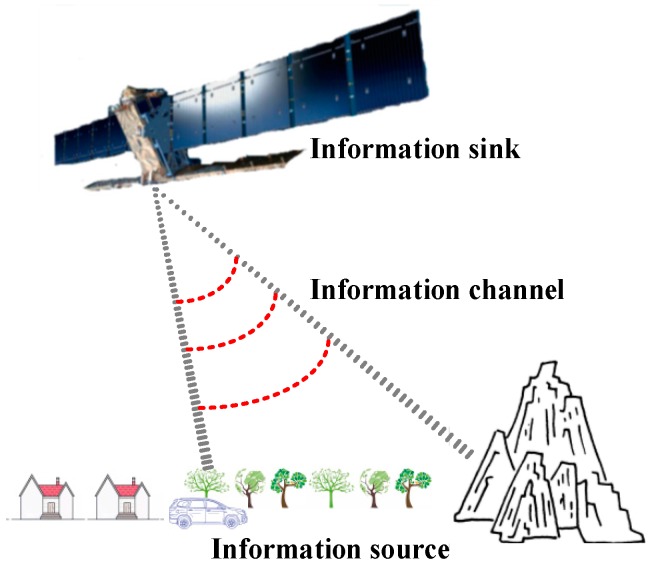
Analogous to the communication system in information theory, the scene, the mapping relation, and the echo correspond to information source, channel, and sink, respectively.

The key factors in the radar imaging system are the scene, the echo, and the mapping relation between the scene and the echo. The mapping relation between the two is influenced by the slant range, the amplitude, phase of the antenna, etc. Analogous to the procedure of information transferring in information theory [39,40], the scene, the mapping relation, and the echo correspond to information source, channel, and sink, respectively, as illustrated in Figure 2. Under this model, if the scene is successfully reconstructed from the echo, it means that the information channel successfully transferred information in a coded way.

**Figure 3:**
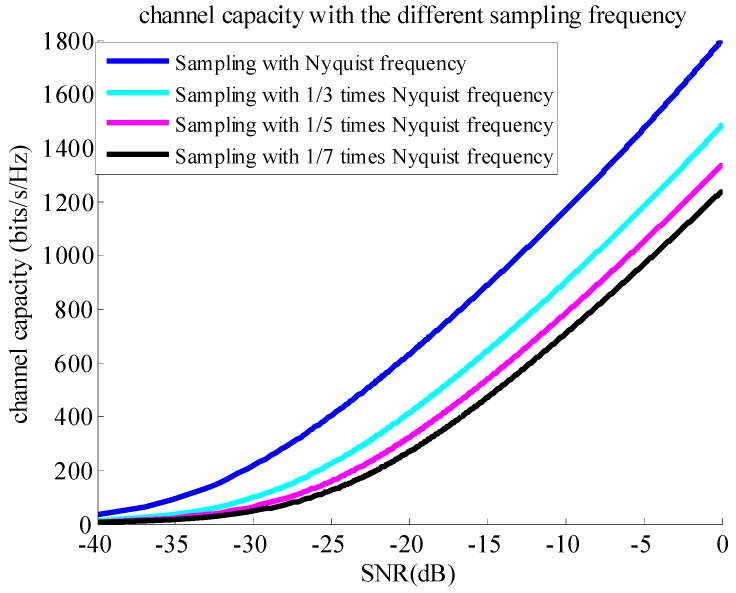
Channel capacity against signal-to-noise ratio (SNR) at different sampling frequencies. As the sampling frequency decreases, channel capacity decreases.

In information theory, the necessary condition of successfully transferring information can be described by noisy-channel coding theorem. This theorem indicates that the transferred information entropy must be smaller than the channel capacity so that all information is nearly error-free transferred to the receiver in a coded way, otherwise, reliable transferring cannot be achieved [40]. Channel capacity, a quantitative index of analyzing information channel, denotes the maximum transferring information entropy with arbitrarily small error probability [40]. Under the information channel model of microwave imaging radar, channel capacity is decided by the reconstructed matrix, and it explains the maximum information entropy of scene is obtained from echo. As done for multiple-input multiple-output (MIMO) communication system, the channel capacity in bits/s/Hz is [40].

(6) $$C\approx \sum_{n=1}^{N}\log_{2}\left(1+SNR\times \beta_{n}^{2}\right),$$

where SNR is the signal-to-noise ratio of the data after range compression for that imaging model based on information channel built on the azimuth dimension, and $\beta_{n}$ is the non-zero singular value of $D_{N\times M}$. Figure 3 illustrates variable curves of channel capacity against SNR at different sampling frequencies. The simulation result shows that channel capacities under the sub-Nyquist sampling method are smaller than those under the Nyquist sampling method, and that smaller sampling frequencies are associated with smaller channel capacities.

Corresponding to the noise-channel coding theorem, the necessary condition of the successful reconstruction is that the channel capacity should be larger than the information entropy of the scene. The reconstructed matrices of Figure 1a,c is the same, so the channel capacity corresponding to Figure 1a,c is also the same. Similarly, the channel capacity corresponding to Figure 1b,d is also the same. However, the information entropy of the scenes in Figure 1a,c is different, for the ocean with several ships is sparser than the urban scene, so that the information entropy of the urban scene is larger than that of the ocean with several ships. The same is true for the scenes in Figure 1b,d. Comparing the result in Figure 1b with that in Figure 1d, the reason for the result in Figure 1d is that the information entropy of the urban scene is larger than the channel capacity under the sub-Nyquist sampling method. Additionally, the simulation results also show that the channel capacity under the Nyquist sampling method is larger than that under the sub-Nyquist sampling method, comparing the result in Figure 1c with that in Figure 1d, as illustrated in Figure 3. Although the scene information entropy is not quantitatively calculated, the above explanation and inference to the results in Figure 1 are logical according to the simulated results. 

Therefore, sub-Nyquist samples in the existing sub-Nyquist SAR system only reconstruct scenes with small sparsity for the reconstructed matrix with small channel capacity, and so the existing sub-Nyquist SAR system has great limitation. In the next section, this limitation will be relieved by pseudo-random space-time modulation.

## 4. Pseudo-Random Space-Time Modulation

For that the reconstructed matrices are decided by the Doppler movement between the moving platform and scene in the existing sub-Nyquist SAR system, the randomness required by the RIP to large value of sparsity may be not enough when the sub-Nyquist sampling rate is certain. Therefore, only changing the movement or sampling method does not improve the reconstruction to large value of sparsity. The reconstructed matrices with good RIP, e.g., random matrix, hold for large values of the sparsity [20,41]. If a scene with large sparsity, e.g., an urban scene, is to be reconstructed with sub-Nyquist samples, it adopts pseudo-random space-time modulation to form the random reconstructed matrix in this paper.

### 4.1. Pseudo-Random Space-Time Modulation

**Figure 4:**
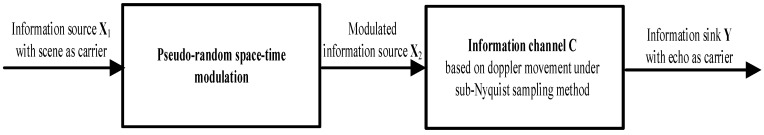
The information channel after pseudo-random space-time modulation. Information channel is based on Doppler movement under the sub-Nyquist sampling method. After pseudo-random space-time modulation, the mutual information between information source X_1_ and information sink Y is increased so that the scene with large value of sparsity is reconstructed.

The RIP implies that randomness plays a major role in the reconstruction. Random measurement waveform or random sub-Nyquist sampling can both yield near-optimal reconstruction with overwhelmingly high probability [42]. The existence of randomness is achieved by pseudo-random modulation regardless of which sub-Nyquist sampling method is adopted. Additionally, the information of slant range is implied in the echo phase [19]. Therefore, this modulation is set to be phase modulation which generates random phase obeying a certain distribution along the azimuthal dimension so that the random reconstructed matrix is formed. This method is essentially the phase modulation, and we named it the pseudo-random space-time modulation in this paper. As illustrated in Figure 4, Information Channel, based on the Doppler movement, uses the modulated scene information as information source, and is echoed as information sink. The procedure involves transferring information in the SAR system.

After this modulation, multiplying the traditional raw data with the complex exponent with variable random phase on space and time generates the new raw data, and the random reconstructed matrix is formed.

### 4.2. Choice of Pseudo-Random Space-Time Modulation

For that Information Channel C is decided by the Doppler movement so that it is immutable at a certain sub-Nyquist sampling frequency, increasing the mutual information between information source X_1_ and information sink Y is to improve this modulation. In this modulation, the phase of complex exponent is a stochastic variable. The distribution of stochastic variable has many distributions, e.g., uniform distribution or Gaussian distribution, and different distributions lead to different reconstruction performance. While the ratio of the maximum to minimum non-zero singular values is small, i.e., *β*_max_/*β*_min_→1, it can transfer more scene information, where *β*_max_ and *β*_min_ are the maximum and minimum non-zero singular values of random modulation matrix, respectively. It can be explained by the restricted isometry constant *δs*. The RIP can be simplified as (1 − *δs*) ≤ *β*_min_^2^ ≤ *β*_max_^2^ ≤ (1 + *δs*), which implies that the smaller restricted isometry constant *δs* leads to the more exact reconstruction, and so it also verifies that the smaller value of *β*_max_/*β*_min_ has the better reconstructed performance from the perspective of restricted isometry constant *δs*. Additionally, singular value decomposition explains that singular value corresponds to the information implied in the matrix and the importance of information is positively correlated with the magnitude of the singular value. Therefore, the maximum non-zero singular value *β*_max_ should be improved to maintain the scene details. During the quantitative analysis of *β*_max_/*β*_min_ and *β*_max_, the mutual coherence coefficient *u* is a widely used variable to be introduced [42], this coefficient explains the maximum similarity between any two columns in $D_{N\times M}$ and is quantitatively denoted as:

(7) $$u=\underset{1\leq i\neq j\leq M}{\max}\frac{\left|\langle D_{i},D_{j}\rangle \right|}{{\left\|D_{i}\right\|}_{2}\cdot {\left\|D_{j}\right\|}_{2}},$$

*β*_max_/*β*_min_ and *β*_max_ are derived to (see Appendix A)

(8) $$\frac{\beta_{\max}}{\beta_{\min}}\leq \frac{1}{{\left(1-\frac{u\cdot \sqrt{M\cdot \left(M-1\right)\cdot \left(1+\frac{1}{M-N+1}\right)}}{1+\sqrt{\frac{M-N}{N\cdot \left(M-1\right)}}}\right)}^{1/2}},$$

and

(9) $${\left(m+m\cdot \sqrt{\frac{M-N}{N\cdot \left(M-1\right)}}\right)}^{1/2}\leq \beta_{\max}\leq {\left(m+m\cdot u\cdot \left(M-1\right)\right)}^{1/2},$$

respectively. In Equation (8), the smaller the mutual coherence *u*, the smaller the ratio *β*_max_/*β*_min_ is and the exacter the whole reconstruction is. Equation (9) denotes that the smaller the mutual coherence *u* is, the smaller *β*_max_ is and the less the scene details are maintained. Therefore, it is undesirable for that the distribution of random phase to choose *u* with a large or small value; rather, it should try to maintain the scene details under guaranteeing the whole error. Equations (8) and (9) indicate the choice of pseudo-random space-time modulation. The sparsity achieved by different modulations can refer to Reference [43].

### 4.3. Carrier of Pseudo-Random Space-Time Modulation

In the pseudo-random space-time modulation, “space” means that different imaging areas have different random phases, and “time” means that different sampling moments have different random phases. This modulation requires the active source of generating the complex exponent with variable random phase on space and time. Then, the question remains as to what is to be taken as the carrier of this modulation.

**Figure 5:**
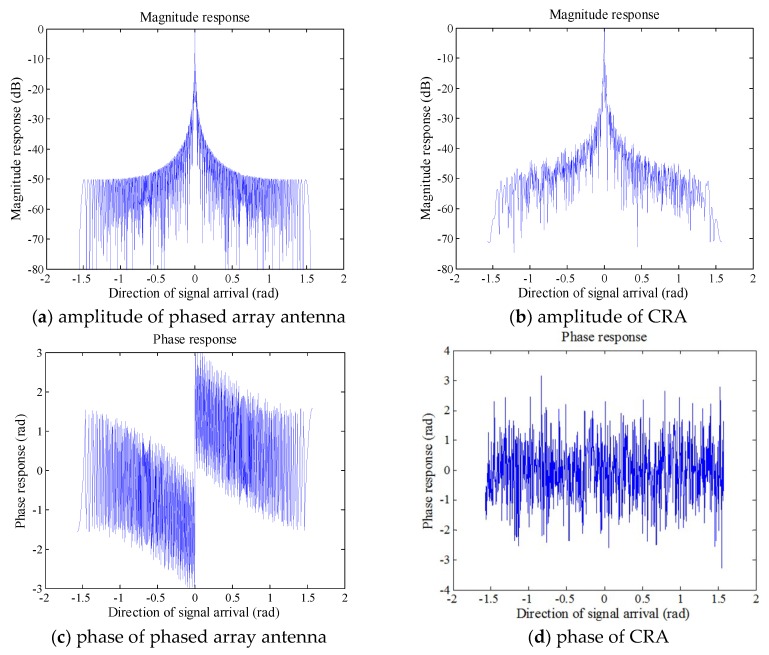
The amplitude and phase patterns of the phased array antenna and compressive reflector antenna (CRA), compared with the linear phase of the phased array antenna; the phase of the CRA is random.

In the SAR system, antenna is an active converter and is hoped to generate spatially and temporally variable random phase. In reality, the antenna with a random phase radiation pattern already exists. The Lincoln Laboratory in the Massachusetts Institute of Technology (MIT) proposed CRA that is an antenna generating a random phase radiation pattern varying in space and time [44,45]. The beamforming of CRA is based on the following two-dimensional coding: (a) spatial coding performed by introducing dielectric or metallic scatterers on the surface of the reflector, and (b) temporal coding through the use of temporal multiplexing of transmitting and receiving horn arrays [46]. The radiation pattern of CRA is shown in Figure 5b,d. The phased array antenna in the traditional SAR system [47] is used for comparison with CRA, and usually generates phase varying linearly as the beam pointing is not random, as illustrated in Figure 5a,c.

The phased array antenna has a high-gain mainlobe and low-gain sidelobe to guarantee the SAR system performance, e.g., the azimuth ambiguity-to-signal ratio (AASR), range ambiguity-to-signal ratio (RASR), and noise-equivalent sigma zero ($NE\sigma^{0}$) [19]. The CRA generates an amplitude radiation pattern with multiple lobes. In this paper, pseudo-random space-time modulation can be achieved by the combination of the phased array antenna and CRA. When transmitting pulses, the traditional antenna works so that the one-way gain is guaranteed. When receiving echoes, CRA works. Although the two-way gain decreases compared with the traditional SAR system, increasing the transmitting power or the technology of depressing sidelobe, e.g., beamforming technology [48], can be adopted to improve the system performance. 

## 5. Sub-Nyquist SAR Based on Pseudo-Random Space-Time Modulation

In this section, sub-Nyquist SAR based on pseudo-random space-time modulation is introduced from four perspectives: the choice of sub-Nyquist sampling method; the echo signal model; the reconstructed method; and the reconstruction performance.

### 5.1. Choice of Sub-Nyquist Sampling Method

Compared with the traditional SAR satisfying the Nyquist sampling theorem, SAR based on pseudo-random space-time modulation works with sub-Nyquist samples. In this paper, sub-Nyquist sampling refers to sampling when the sampling frequency is smaller than the Nyquist sampling frequency in this paper. There are two typical methods for sub-Nyquist sampling method: random sub-Nyquist sampling, e.g., random equivalent sampling (RES) [49,50] and multicoset sampling [51,52], and uniform sub-Nyquist sampling [53,54]. The random sub-Nyquist sampling method involves sampling at an arbitrary sampling interval, and the uniform sub-Nyquist sampling method involves sampling at a certain sampling interval.

**Figure 6:**
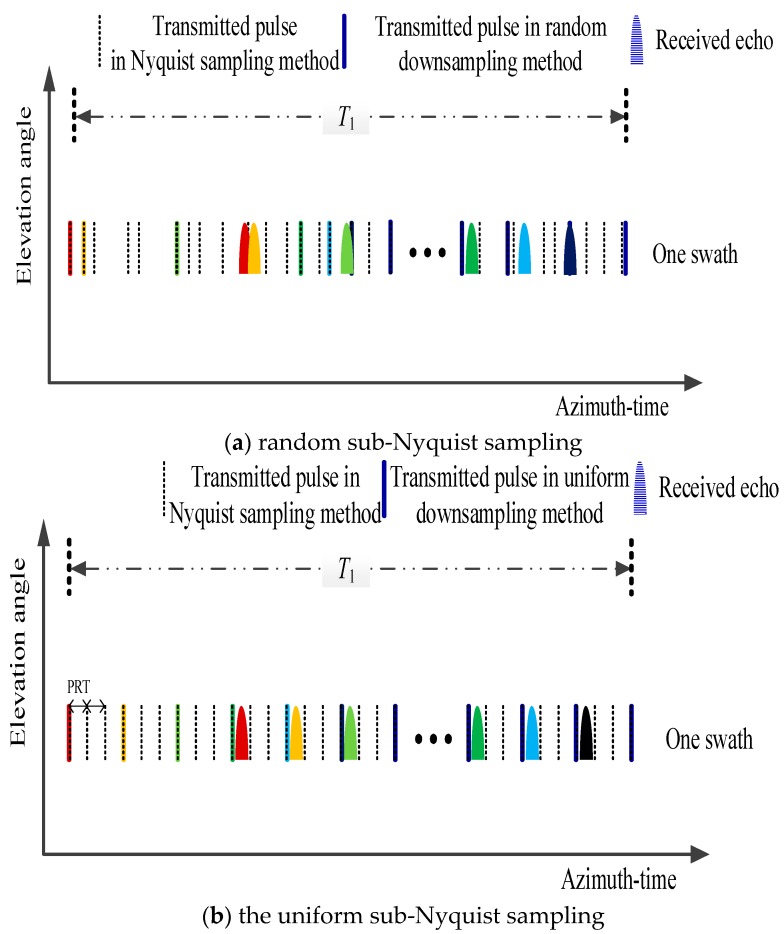
Illustration of sub-Nyquist sampling method for sampling duration *T*_1_. The dashed line denotes the Nyquist sample. The solid line denotes the sub-Nyquist sample in random and uniform sub-Nyquist sampling method randomly and uniformly selects from dashed line, respectively. (**a**) random sub-Nyquist sampling: the conflict between transmitted pulse and receiving echo. (**b**) uniform sub-Nyquist sampling: echo is completely received and has no conflict with the transmitted pulse.

Because the observational distance is far in the spaceborne SAR system, the time interval between transmitting and receiving is several times the Nyquist pulse repetition time (PRT). The system design should ensure that the echo is completely received and avoids the nadir echo. In the random sub-Nyquist sampling method, the received echo may overlap with the transmitted pulse or the nadir echo. Unless a SAR system adopts one way to avoid the overlapping, the random sub-Nyquist sampling method is not feasible, as illustrated in Figure 6a. Considering that it should avoid the overlapping between transmitting and receiving and that the pseudo-random space-time modulation already guarantees the existence of randomness required by reconstruction, sub-Nyquist samples can be achieved by the uniform sub-Nyquist sampling method in this paper.

Although the uniform sub-Nyquist sampling on the azimuth dimension is sparse, its azimuthal spectrum span is equal to the entire Doppler bandwidth. This lays a good foundation for image reconstruction without the loss of resolution. Additionally, because sub-Nyquist sampling reduces the SNR in Equation (6), the transmitting power can be increased to guarantee information capacity.

### 5.2. Echo Signal Model after Pseudo-Random Space-Time Modulation

Traditional echo model, it only has the antenna amplitude weighting, not the antenna phase weighting for which the traditional antenna generates a linear phase, and it has little effect on the echo. Unlike with the traditional antenna, the phase is random, so that the phase weighting must be considered in the echo signal model. Therefore, in order to analyze SAR based on the pseudo-random space-time modulation, the corresponding echo signal model should be set up. Different scene positions corresponding to different beam pointing have different time-varying random phase weighting. During the physical formation process of this modulated system based on CRA, it should distinguish the key factors affecting the echo signal, e.g., platform movement, antenna amplitude and phase, and the transmitting signal. After the pseudo-random space-time modulation, the SAR echo for point targets when transmitting the same signal with Equation (1) is denoted as

(10) $$\begin{array}{ll} p_{0}\left(\tau ,\eta \right)= & \sum_{x_{i},y_{i}}^{}\sigma_{i}W_{i}\left(\tau ,\eta \right)rect\left\{\left(\tau -\frac{2R_{i}\left(\eta \right)}{c}\right)/T_{r}\right\}\cdot \exp\left\{j\varphi_{i}\left(\eta \right)\right\}\\ & \;\exp\left\{j\pi K_{r}{\left[\tau -\frac{2R_{i}\left(\eta \right)}{c}\right]}^{2}\right\}\cdot \exp\left\{-j4\pi \frac{R_{i}\left(\eta \right)}{\lambda }\right\}+n\left(\tau ,\eta \right) \end{array}$$

where $\varphi_{i}\left(\eta \right)$ is the random phase generated by CRA corresponding to the target at (*x_i_*,*y_i_*). The different scene position (*x_i_*,*y_i_*) corresponding to different beam pointing has different time-varying random phase $\varphi_{i}\left(\eta \right)$.

### 5.3. Reconstructed Method after Pseudo-Random Space-Time Modulation

As mentioned in Section 2, no matter which algorithm is adopted, the reconstructed algorithm includes three steps: the range compression, RCMC, and the azimuth compression. For the range compression, when the transmitted signal is the linear frequency modulated (LFM) signal, the range signal can be compressed based on matched filtering (MF). Most of the already existing algorithms correct range migration in the azimuth frequency domain. When targets on the same range cell have the same range migration in the azimuth frequency domain, correcting the range migration of one target is equivalent to correcting that of targets on the same range cell so that correcting range migration in the frequency domain is efficient [19]. For azimuth compression in the time or frequency domain, MF is also often adopted when it at least has Nyquist samples. However, in the sub-Nyquist SAR based on the pseudo-random space-time modulation, the echo signal is sampled at a sub-Nyquist frequency, so that the azimuth compression based on MF is no longer applicable. Based on the analysis in Section 2, the azimuth signal with sub-Nyquist samples can be recovered by the optimization equation of Equation (5). The demonstration of the reconstructed process is given as follows:

(1) Range Compression

Because the pseudo-random space-time modulated SAR system transmits a LFM signal, range compression can still adopt MF. Assuming that

(11) $$H\left(f_{\tau }\right)=rect\left(\frac{f_{\tau }}{\left|K_{r}\right|T_{r}}\right)\exp\left(j\pi \frac{f_{\tau }{}^{2}}{K_{r}}\right),$$

is matched filter of the range dimension in the frequency domain, the signal after range compression is

(12) $$\begin{array}{ll} p_{rc}\left(\tau ,\eta \right)= & {\mathrm{IFFT}}_{\tau }\{p_{0}(f_{\tau },\eta )H(f_{\tau })\}\\ & \sum_{x_{i},y_{i}}^{}\sigma_{i}W_{i}\left(\tau ,\eta \right)T_{r}\cdot \sin c\{K_{r}T_{r}[\tau -\frac{2R_{i}\left(\eta_{c_{i}}\right)}{c}]\}\cdot \\ & \exp\left\{j\varphi_{i}\left(\eta \right)\right\}\cdot \exp\left\{-\frac{j4\pi R_{i}\left(\eta \right)}{\lambda }\right\}+n\left(\tau ,\eta \right) \end{array}$$

where $P_{0}\left(f_{\tau },\eta \right)$ is the Fourier transform of the signal $p_{0}\left(\tau ,\eta \right)$ on the range dimension, ${\mathrm{IFFT}}_{\tau }\left\{\cdot \right\}$ is the inverse Fourier transform on the range dimension, and sinc(·) is the sinc function.

(2) Range Cell Migration Correction (RCMC)

In the traditional SAR system, the azimuthal signal is nearly an LFM signal, and the traditional algorithms, e.g., range Doppler algorithm (RDA) or chirp scaling, efficiently correct range migration in the azimuthal frequency domain [19]. After pseudo-random space-time modulation, the azimuthal signal in the frequency domain does not have the explicit expression. The RCMC is implemented in the azimuth time domain in this paper. The procedure of the RCMC is as follows: (1) firstly, it should choose the imaging area at least guaranteeing to contain the observed area in the raw data. Cell division of the imaging area should be smaller than or equal to the resolution of the raw data. When the cell of the imaging area is divided more precisely, the resolution will not change, and this seriously affects the imaging efficiency. Therefore, the cell division of the imaging area should be slightly smaller than or equal to the resolution of the raw data; (2) it takes the ascending sampling to the raw data along the range dimension and calculates the slant range from each division cell of the imaging area to radar at each sampling time. Then, find the position of each division cell on the ascending sampling range cell at each sampling moment according to the time delay calculated by the slant range and take the data on this position; (3) calculate the sum on the data of division cell on the same nearest slant range. After this, the RCMC is finished. After application of the RCMC, the signal is denoted by

(13) $$\begin{array}{ll} p_{rcm}\left(\tau ,\eta \right)= & \sum_{x_{i},y_{i}}^{}\sigma_{i}W_{i}\left(\tau ,\eta \right)T_{r}\cdot \sin c\{K_{r}T_{r}[\tau -\frac{2R_{i}\left(\eta_{c_{i}}\right)}{c}]\}\cdot \\ & \exp\left\{j\varphi_{i}\left(\eta \right)\right\}+n\left(\tau ,\eta \right) \end{array}$$

(3) Azimuth Compression

At the range sampling moment $\tau_{0}$, Equation (13) can be expressed corresponding to each sampling moment $\eta_{n}$ as

(14) $$p_{N\times 1}=\left[\begin{array}{c} p_{rcm}(\tau_{0},\eta_{1})\\ p_{rcm}(\tau_{0},\eta_{2})\\ \vdots \\ p_{rcm}(\tau_{0},\eta_{n})\\ \vdots \\ p_{rcm}(\tau_{0},\eta_{N}) \end{array}\right]=\varphi_{N\times M}\sigma_{M\times 1}+n_{N\times 1},$$

where $\sigma_{M\times 1}={\left[\sigma_{1},\sigma_{2},\cdots ,\sigma_{M}\right]}^{T}$, $n_{N\times 1}={\left[n\left(\tau_{0},\eta_{1}\right),n\left(\tau_{0},\eta_{2}\right),\cdots ,n\left(\tau_{0},\eta_{N}\right)\right]}^{T}$, and $\varphi_{N\times M}=\{W_{i}\left(\tau_{0},\eta_{n}\right)\cdot T_{r}\cdot \sin c\left\{K_{r}T_{r}\left[\tau_{0}-\frac{2R_{i}\left(\eta_{c_{i}}\right)}{c}\right]\right\}\cdot {\exp\left[j\varphi_{i}\left(\eta_{n}\right)\right]\}}_{n=1,i=1}^{N,M}$.

Because the samples are achieved with the sub-Nyquist frequency, the optimization equation of Equation (14) without subscript is as follows:

(15) $$\hat{\sigma }=\underset{\sigma }{\arg\min}\left\{{\left\|p-\varphi \sigma \right\|}_{2}^{2}+\gamma {\left\|\sigma \right\|}_{1}\right\},$$

where $\hat{\sigma }$ is the reconstructed result at the range sampling moment $\tau_{0}$. The imaging area is reconstructed at all the range sampling moments after the RCMC.

### 5.4. Reconstructed Performance

The reconstruction equation without subscript is rewritten as:

(16) p=φσ+n,

Usually, the components of noise n are approximated as a Gaussian distribution with zero-mean and variance $\sigma_{n}^{2}$ [30]. The scene backscattering cross-section is affected by the observation incidence angle, wavelength, surface structure, etc., and many effect factors are not totally accounted for by a simple deterministic data model [55]. The prior distribution of σ is approximate from SAR image even although speckle noise exists. The statistics of SAR image have been investigated under the assumption of Gaussian statistics with zero-mean and variance $\sigma_{x}^{2}$ for the backscattering cross-section [56,57]. Under the above assumption about the noise n and the scene σ, it follows that:

(17) $$\left\{ \begin{array}{l} p_{n}\left(n\right)=p_{p|\sigma }\left(p|\sigma \right)=\frac{1}{\sqrt{2\pi }\sigma_{n}}\exp\left\{-\frac{{\left\|p-\varphi \sigma \right\|}_{2}^{2}}{2\sigma_{n}^{2}}\right\}\\ p_{\sigma }\left(\sigma \right)=\frac{1}{\sqrt{2\pi }\sigma_{x}}\exp\left\{-\frac{{\left\|\sigma \right\|}_{2}^{2}}{2\sigma_{x}{}^{2}}\right\} \end{array}\right.,$$

The knowledge of the reconstruction performance based on CS is interesting. Since the CS recovery algorithm is an estimation method, classical point target evaluation system (e.g., 3 dB resolution, integral side lobe ratio (ISLR), and peak side lobe ratio (PSLR)) are not available in the SAR system based on CS. For a given scene σ, the reconstruction performance can be evaluated by the mean square error (MSE) $E\left\{{\left(\hat{\sigma }-\sigma \right)}^{2}\right\}$, where $\hat{\sigma }$ is the reconstruction result. In the Bayesian framework, the estimation error is simplified as (see Appendix B)

(18) $$E\left\{{\left(\hat{\sigma }-\sigma \right)}^{2}\right\}\geq trace\left[{\left(\frac{\varphi^{H}\varphi }{\sigma_{n}^{2}}+\frac{I}{\sigma_{x}^{2}}\right)}^{-1}\right],$$

where trace(·) denotes the trace of a matrix and I is the unit matrix.

## 6. Validation and Analysis

**Figure 7:**
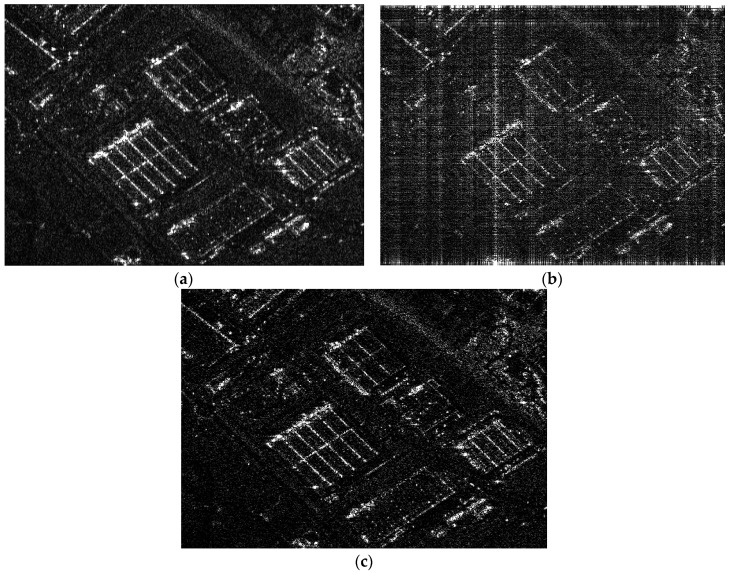
(**a**) Reconstructed result from Nyquist samples; (**b**) Reconstructed result from sub-Nyquist samples; (**c**) Reconstructed result from sub-Nyquist samples after the pseudo-random space-time modulation.

Sub-Nyquist SAR based on pseudo-random space-time modulation is simulated and compared with the traditional SAR with Nyquist samples. The corresponding simulated parameters are listed in Table 1. Figure 7c shows that the urban scene is successfully reconstructed, and its main details are maintained after pseudo-random space-time modulation compared with the result in Figure 7b. Although the random sub-Nyquist sampling method is adopted in Figure 7b and the uniform sub-Nyquist sampling method in Figure 7c, the average PRFs are both 278 Hz.

**Figure 8:**
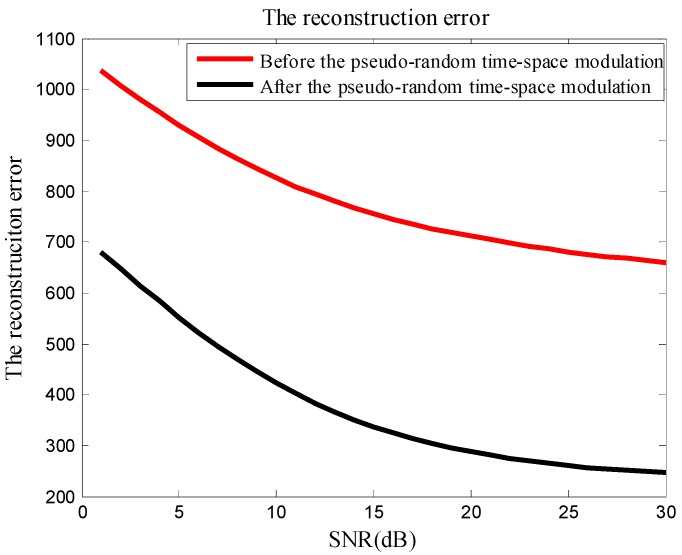
The reconstruction error before and after the pseudo-random time-space modulation.

According to the optimization reconstruction performance in Equation (18), Figure 8 shows that the reconstruction error decreases after pseudo-random space-time modulation.

**Figure 9:**
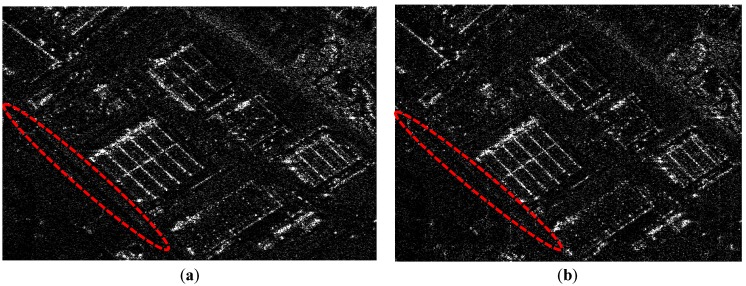

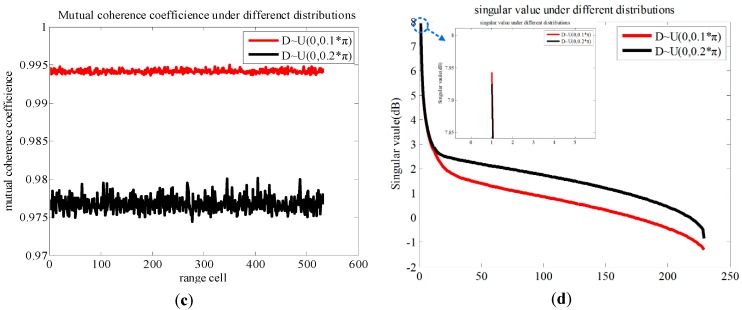
Simulated results of (**a**,**b**) reconstructed scene, (**c**) mutual coherence coefficient, and (**d**) singular value.

The simulated results in Figure 9 verify the decision to indicate the choice of pseudo-random space-time modulation in Section 4. As previously mentioned, the phase of complex exponent generated by CRA is a stochastic variable. The simulation chooses that the phase has a uniform distribution with different variances to compare and analyze. After pseudo-random space-time modulation, results in Figure 9a,b are successfully reconstructed with sub-Nyquist samples compared to Figure 1d. Results of Figure 9a,b are simulated when the phase of pseudo-random space-time modulation obeys a uniform distribution with different variances. Results of Figure 9c,d denote the curves of mutual coherence coefficient, and singular value amplitude under two distributions. For the convenience of description, assuming the mutual coherence coefficient, the maximum singular value and the minimum singular value under two different random distributions are denoted by *u_u_*, *u_g_*, *β*_max_*u*_, *β*_max_*g*_, *β*_min_*u*_, and *β*_min_*g*_, respectively. As illustrated in Figure 9, *u_u_* is larger than *u_g_* in Figure 9c, *β*_max_*u*_ and *β*_max_*u*_/*β*_min_*u*_ are larger than *β*_max_*g*_ and *β*_max_*g*_/*β*_min_*g*_ in Figure 9d, respectively, and more road details are maintained in the red ellipsoid of Figure 9a compared with Figure 9b. It is consistent with Equations (8) and (9) that random phase distribution with large mutual coherence leads to many scene details under guaranteeing the whole error. Pseudo-random space-time can improve the imaging performance, and Equations (8) and (9) indicate the choice of the modulation.

## 7. Conclusions

This paper presents sub-Nyquist SAR based on pseudo-random space-time modulation. To achieve this system, CRA with high-sensing capacity was adopted to be a carrier of pseudo-random space-time modulation. The following two major findings have been obtained:

(1) To explain the reason for the constraint on the scene sparsity in the traditional sub-Nyquist SAR, this paper establishes an information channel model of microwave imaging radar based on information theory. Under this model, noisy-channel coding theorem indicates the necessary condition of the successful reconstruction and explains the reason for this constraint.

(2) To relieve the constraint on the scene sparsity so that the scene with large sparsity can be reconstructed, this paper proposes sub-Nyquist SAR based on pseudo-random space-time modulation. This modulation is the phase modulation of the traditional raw data and it can reduce the mutual information of successive measurements so that more scene information can be obtained.

The proposed method is applied not only to the strip mapping mode but also to other modes such as sliding spotlight and terrain observation with progressive scan (TOPS) modes. Our future research will focus on a wide-swath SAR system based on pseudo-random space-time modulation.

## Figures and Tables

**Table 1:** Simulation parameters.

Parameter	Values of the Nyquist Sampling Method	Values of the Sub-Nyquist Sampling Method
Orbital height (km)	693	693
Wavelength (m)	0.0555	0.0555
Pulse width (µs)	50	50
Antenna height (m)	8.93	8.93
Signal bandwidth (MHz)	100	100
The sampling frequency (MHz)	110	110
Incidence angles (°)	38.73–43.50	38.73–43.50
(Average) pulse repetition frequency (PRF) (Hz)	1946	278

## Appendix A

Assuming that $A_{M\times M}=D_{N\times M}{}^{H}D_{N\times M}$ and $m=\bar{{\left\|D_{i}\right\|}_{2}^{2}},i\in \left\{1,2,\cdots ,M\right\}$. For that $A_{M\times M}$ is Hermitian matrix and N<M, $A_{M\times M}$ has N non-zero real eigenvalues $\alpha_{1},\alpha_{2},\cdots ,\alpha_{N}$ in descending numerical order, M−N zero eigenvalues $\alpha_{N+1}=\alpha_{N+2}=\cdots =\alpha_{M}=0$. The singular value of $D_{N\times M}$ is $\beta_{i}=\sqrt{\alpha_{i}}$. The trace of $A_{M\times M}$ and $A_{M\times M}{}^{2}$ are

(A1) $$trace(A_{M\times M})=\sum_{n=1}^{N}\alpha_{n}=M\cdot m,$$

(A2) $$\begin{array}{l} trace(A_{M\times M}{}^{2})=\sum_{n=1}^{N}\alpha_{n}{}^{2}=\sum_{i=1}^{M}\sum_{j=1}^{M}{\left|\langle D_{i},D_{j}\rangle \right|}^{2}\\ =M\cdot m^{2}+2\cdot \sum_{i=1}^{M}\sum_{j=i+1}^{M}{\left|\langle D_{i},D_{j}\rangle \right|}^{2}\\ \leq M\cdot m^{2}+M\cdot \left(M-1\right)\cdot u^{2}\cdot m^{2} \end{array}$$

Cauchy-Schwarz inequality shows

(A3) $$\sum_{n=1}^{N}\alpha_{n}{}^{2}\geq \frac{{\left(\sum_{n=1}^{N}\alpha_{n}\right)}^{2}}{N}=\frac{M^{2}\cdot m^{2}}{N},$$

Combining Equations (A2) and (A3) gives:

(A4) $$\frac{M^{2}\cdot m^{2}}{N}\leq trace(A_{M\times M}{}^{2})\leq M\cdot m^{2}+M\cdot \left(M-1\right)\cdot u^{2}\cdot m^{2},$$

Assuming that $b=\sqrt{trace\left(A_{M\times M}{}^{2}\right)/M-m^{2}}$, Equation (A4) is substituted into b:

(A5) $$m\cdot \sqrt{\frac{M-N}{N}}\leq b\leq m\cdot u\cdot \sqrt{M-1},$$

Reference [58] indicates

(A6) $$m+b/\sqrt{M-1}\leq \alpha_{\max}\leq m+b\cdot \sqrt{M-1},$$

(A7) $$\alpha_{k}-\alpha_{l}\leq b\cdot \sqrt{M\cdot \left(\frac{1}{k}+\frac{1}{M-l+1}\right)},1\leq k\leq l\leq M,$$

Equation (A5) is substituted into Equation (A6),

(A8) $${\left(m+m\cdot \sqrt{\frac{M-N}{N\cdot \left(M-1\right)}}\right)}^{1/2}\leq \beta_{\max}\leq {\left(m+m\cdot u\cdot \left(M-1\right)\right)}^{1/2},$$

When k=1 and l=N, Equation (A7) is simplified as

(A9) $$\alpha_{1}-\alpha_{N}\leq b\cdot \sqrt{M\cdot \left(1+\frac{1}{M-N+1}\right)},$$

It divides by $\alpha_{1}$ at two sides of Equation (A9) and applies the range of $\alpha_{1}$,

(A10) $$1-\frac{\alpha_{N}}{\alpha_{1}}\leq \frac{b\cdot \sqrt{M\cdot \left(1+\frac{1}{M-N+1}\right)}}{\alpha_{1}}\leq \frac{b\cdot \sqrt{M\cdot \left(1+\frac{1}{M-N+1}\right)}}{m+m\cdot \sqrt{\frac{M-N}{N\cdot \left(M-1\right)}}},$$

The ratio of the maximum to minimum eigenvalue $\alpha_{1}/\alpha_{N}$ is achieved by Equation (A10)

(A11) $$\frac{\alpha_{1}}{\alpha_{N}}\leq \frac{1}{1-\frac{b\cdot \sqrt{M\cdot \left(1+\frac{1}{M-N+1}\right)}}{m+m\cdot \sqrt{\frac{M-N}{N\cdot \left(M-1\right)}}}},$$

$\beta_{1}/\beta_{N}$ is further achieved by applying the range of b

(A12) $$\frac{\beta_{1}}{\beta_{N}}\leq \frac{1}{{\left(1-\frac{u\cdot \sqrt{M\cdot \left(M-1\right)\cdot \left(1+\frac{1}{M-N+1}\right)}}{1+\sqrt{\frac{M-N}{N\cdot \left(M-1\right)}}}\right)}^{1/2}},$$

## Appendix B

Assuming that the vector σ is a random variable vector, the mean square error (MSE) of the estimation $\hat{\sigma }$ in a Bayesian framework is denoted by Reference [59]

(A13) $$\begin{array}{ll} \mathrm{MSE}\left(\hat{\sigma }\right) & =\sum_{m=1}^{M}E\left\{{\left({\hat{\sigma }}_{m}-\sigma_{m}\right)}^{2}\right\}\\ & \geq trace\left({\left\{-E\left[\frac{\partial^{2}\ln p_{p,\sigma }\left(p,\sigma \right)}{\partial \sigma_{m1}\partial \sigma_{m2}}\right]\right\}}^{-1}\right)\\ & =trace\left({\left\{-E\left[\frac{\partial^{2}\ln p_{p/\sigma }\left(p/\sigma \right)}{\partial \sigma_{m1}\partial \sigma_{m2}}\right]-E\left[\frac{\partial^{2}\ln p_{\sigma }\left(\sigma \right)}{\partial \sigma_{m1}\partial \sigma_{m2}}\right]\right\}}^{-1}\right) \end{array}$$

where ${\hat{\sigma }}_{m}$ and $\sigma_{m}$ are the *m*th element of $\hat{\sigma }$ and σ, respectively. trace(·) denotes the trace of a matrix. $p_{p,\sigma }\left(p,\sigma \right)$, $p_{p/\sigma }\left(p/\sigma \right)$ and $p_{\sigma }\left(\sigma \right)$ are the joint probability density function of the vector p and σ, the conditional probability density function of the vector p and σ, the prior probability density function of the vector σ, respectively. The data information matrix $J_{D}$ and the prior information matrix $J_{P}$ represent $-E\left[\frac{\partial^{2}\ln p_{p/\sigma }\left(p/\sigma \right)}{\partial \sigma_{m1}\partial \sigma_{m2}}\right]$ and $-E\left[\frac{\partial^{2}\ln p_{\sigma }\left(\sigma \right)}{\partial \sigma_{m1}\partial \sigma_{m2}}\right]$, respectively.

The following analyzes the Bayesian information matrix $J_{B}=J_{D}+J_{P}$ from these two information matrices:

(1) the calculation of the data information matrices $J_{D}$:
  (A14) $$\begin{array}{l} J_{D}=-E\left[\frac{\partial^{2}\ln p_{p/\sigma }\left(p/\sigma \right)}{\partial \sigma_{m1}\partial \sigma_{m2}}\right]=E\left[\frac{1}{2\sigma_{n}{}^{2}}\cdot \frac{\partial {\left\|p-\varphi \sigma \right\|}_{2}^{2}}{\partial \sigma_{m1}\partial \sigma_{m2}}\right]\\ =E\left\{\frac{1}{2\sigma_{n}{}^{2}}\cdot \frac{\partial }{\partial \sigma_{m1}\partial \sigma_{m2}}\left[p^{H}p-p^{H}\varphi \sigma -\sigma^{H}\varphi^{H}\sigma +\sigma^{H}\varphi^{H}\varphi \sigma \right]\right\}\\ =E\left\{\frac{1}{2\sigma_{n}{}^{2}}\cdot \frac{\partial }{\partial \sigma_{m2}}\left[2\varphi^{H}\varphi \sigma -2\varphi^{H}p\right]\right\}\\ =\frac{\varphi^{H}\varphi }{\sigma_{n}{}^{2}} \end{array}$$
(2) the calculation of the prior information matrix $J_{P}$:
  (A15) $$\begin{array}{l} J_{P}=-E\left[\frac{\partial^{2}\ln p_{\sigma }\left(\sigma \right)}{\partial \sigma_{m1}\partial \sigma_{m2}}\right]=E\left[\frac{1}{2\sigma_{x}{}^{2}}\cdot \frac{\partial {\left\|\sigma \right\|}_{2}^{2}}{\partial \sigma_{m1}\partial \sigma_{m2}}\right]\\ =\frac{I}{\sigma_{x}{}^{2}} \end{array}$$
  where I is the unit matrix. Substituting Equations (A13) and (A14) into Equation (A15), we obtain
  (A16) $$E\left\{{\left(\hat{\sigma }-\sigma \right)}^{2}\right\}\geq trace\left[{\left(\frac{\varphi^{H}\varphi }{\sigma_{n}^{2}}+\frac{I}{\sigma_{x}^{2}}\right)}^{-1}\right],$$
